# Sedation and Anesthesia of Galapagos (*Chelonoidis nigra*), Aldabra (*Aldabrachelys gigantea*), and African Spurred Tortoises (*Centrochelys sulcata*): A Retrospective Review (2009–2019)

**DOI:** 10.3390/ani11102920

**Published:** 2021-10-09

**Authors:** Rachel C. Turner, Bonnie J. Gatson, Jorge A. Hernandez, Amy B. Alexander, Copper Aitken-Palmer, Alessio Vigani, Darryl J. Heard

**Affiliations:** 1 The Birmingham Zoo, 2630 Cahaba Road, Birmingham, AL 35223, USA; 2 Safe Harbor Veterinary Anesthesia Support LLC, 10205 SW 75th Way, Gainesville, FL 32608, USA; bonnie.gatson@safeharboranesthesia.com; 3 Department of Large Animal Clinical Sciences, University of Florida College of Veterinary Medicine, 2015 SW 16th Avenue, Gainesville, FL 32610, USA; hernandezja@ufl.edu; 4 Department of Comparative, Diagnostic, and Population Medicine, University of Florida College of Veterinary Medicine, 2015 SW 16th Avenue, Gainesville, FL 32610, USA; alexandera@ufl.edu (A.B.A.); heardd@ufl.edu (D.J.H.); 5 Chicago Zoological Society, Brookfield Zoo, 3300 Golf Road, Brookfield, IL 60513, USA; copper.aitken-palmer@czs.org; 6 Department fur Kleintiere, Vetsuisse Faculty, University of Zurich, Winterhurerstrasse 258c, 8057 Zurich, Switzerland; alessio.vigani@gmail.com

**Keywords:** Aldabra, African-spurred, anesthesia, Galapagos, tortoise

## Abstract

**Simple Summary:**

Anesthesia is often required for the medical management of large tortoise species, but little has been published regarding effective anesthetic regimens for these species. The purpose of this study was to review anesthetic regimens that have been used safely and effectively in Galapagos (*Chelonoidis nigra*), Aldabra (*Aldabrachelys gigantea*), and African spurred (*Centrochelys sulcata*) tortoises, with the aim of improving medical management.

**Abstract:**

Tortoises belong to the taxonomic family *Testudinidae,* which is considered one of the most imperiled families of the order *Testudines*. Anesthesia is often required for the medical and surgical management of large tortoises. The objectives of this retrospective study were to review drug regimens used to successfully anesthetize Galapagos (*Chelonoidis nigra),* Aldabra *(Aldabrachelys gigantea)* and African spurred (*Centrochelys sulcata)* tortoises, and to compare the times to effect and to extubation in tortoises administered different premedication protocols. Anesthetic records of giant tortoises admitted to the University of Florida College of Veterinary Medicine between January 2009 and December 2019 were reviewed. A total of 34 tortoises (six Aldabra, 23 Galapagos, and five African spurred) were included, resulting in 64 anesthetic events. Frequently used premedication protocols included an α_2_-adrenergic agonist and ketamine combined with either midazolam (group α_2_−adrenergic agonist, midazolam, ketamine, AMK; *n* = 34), a μ-opioid receptor agonist (group α_2_−adrenergic agonist, μ-opioid receptor agonist, ketamine, AOK; *n* = 13), or a μ−opioid receptor agonist and midazolam (group α_2_−adrenergic agonist, midazolam, μ-opioid receptor agonist, ketamine, AMOK; *n* = 10). Inhalant anesthetics (isoflurane, *n* = 21; sevoflurane, *n* = 23) were frequently used for maintenance of anesthesia following premedication. Out of the 34 total tortoises, 22 had only one anesthetic event, five had two anesthetic events, three had three anesthetic events, and four had four or more anesthetic events. Few adverse effects were observed and there was no mortality reported during the peri-anesthetic period. Sedation and general anesthesia of giant tortoises can be successfully performed with a combination of an α_2_-adrenergic agonist and ketamine in combination with midazolam and/or a μ−opioid receptor agonist.

## 1. Introduction

Physical examination and diagnostic evaluation can be difficult in conscious giant tortoises because of their unique anatomy, large size, and behavior. Consequently, sedation and anesthesia are often essential to perform medical and surgical procedures. Few published studies describe successful anesthesia of the largest tortoise species. Techniques for anesthesia and analgesia have been described for Galapagos tortoises (*Chelonoidis nigra)* undergoing surgical sterilization. These regimens included ketamine and medetomidine in females, and intrathecal lidocaine in males [1,2]. Medetomidine and ketamine have also been used to anesthetize Aldabra tortoises (*Aldabrachelys gigantea)* [3]. Anesthesia of African spurred tortoises (*Centrochelys sulcata*) has been reported using combinations of midazolam, medetomidine, ketamine and morphine [4,5]. All of these reports provide limited information on time to sedation and recovery and do not describe any potential adverse effects.

The main objectives of this study were to describe anesthetic protocols used in three giant tortoise species (Aldabra, Galapagos, and African spurred), and to compare the dosages used between these species. 

## 2. Materials and Methods

As a retrospective clinical study, approval from the Institutional Animal Care and Use Committee of the University of Florida was not required. Data retrieval was limited to complete records of anesthesia of Galapagos, Aldabra, and African spurred tortoises that underwent general anesthesia at the University of Florida College of Veterinary Medicine from 2009 to 2019. Records were considered complete if all drug dosages were recorded. No records were excluded from our analysis. Digital records were identified by searching for all tortoise anesthetic events from this time period, and then sorting them by species. The data was retrieved and organized by one author using Microsoft Excel software. Information retrieved from medical and anesthesia records included species, sex, age (as reported by the owner or caretaker), weight, presenting complaint, preanesthetic hematologic analysis, comorbidities, medications, and any anesthetic event within the past seven days. Anesthetic drugs used for premedication, induction, and maintenance, antibiotics, fluids, anesthetic reversal agents, and route of administration of all medications were also recorded. Premedication protocols were categorized based on drug class: α_2_-adrenergic agonists, benzodiazepines, dissociatives, and μ−opioid receptor agonists. Time to intubation and type and size of the endotracheal tube were recorded for animals that underwent endotracheal intubation. Time to intubation was defined as the time from administration of the first premedication combination to placement of the endotracheal tube. In addition, the mode of ventilation (spontaneous, manual or both) was recorded. Monitoring devices, recorded physiologic variables, and any peri-anesthetic complications were also noted. 

Time to effect (minutes) was defined as that from initial drug administration to either the start of maintenance anesthesia or when sedation was adequate to facilitate the intended procedure. Time to extubation (minutes) was defined as either time from discontinuation of inhalant anesthetic gas to removal of the endotracheal tube, or time from the end of the procedure to removal of the endotracheal tube for patients that were not administered inhalants. 

To accomplish the main study objective, data from all 64 anesthesia episodes were included in the analysis. Descriptive statistics were calculated from the signalment and historical data, which included species, age, sex, and body weight. Drug dosages are reported as mean with a dosing range if appropriate, and all time variables are reported as median [interquartile range (IQR)]. Ages and weights are reported as mean ± standard deviation (range). The non-parametric Kruskal–Wallis test was used to test the null hypothesis that dosages of drugs administered were not significantly different between species. In addition, a power analysis was performed to evaluate the strength of these comparisons. Analyses were performed using Sigmaplot Version 14.0 (Systat Software Inc., San Jose, CA, USA).

## 3. Results

The 64 anesthetic events included six Aldabra tortoises (aged 32 ± 15 (16–65) years, weighing 139 ± 32 (52–210) kg), 23 Galapagos tortoises (aged 42 ± 28 (6–85) years weighing 119 ± 56 (13.5–250) kg), and five African spurred tortoises (aged 11 ± 7 (1–20) years, weighing 22 ± 23 (0.5–57.7) kg). There were 18 females, 14 males and two that were undetermined sex. Indications for anesthesia included computed tomography (*n =* 29), esophageal feeding tube placement (*n =* 10), radiography (*n =* 10), wound care (*n =* 10), biopsy (*n =* 8), venipuncture (*n =* 6), echocardiography (*n =* 5), endoscopy (*n =* 5), cloacal prolapse repair and reduction (*n=* 3), fine needle aspiration (*n =* 3), tracheal or bronchiolar lavage (*n =* 3), ultrasonography (*n =* 3), carapace fracture repair (*n =* 2), cystolithotomy (*n =* 2), and one each for bronchoscopy, cloacolith removal, surgical exploration of an inguinal mass, and tail amputation. Many of the tortoises had multiple procedures performed during a single anesthetic event. Out of the 34 total tortoises, 22 had only one anesthetic event, five had two anesthetic events, three had three anesthetic events, and four had four or more anesthetic events. 

Drugs and dosages, route of administration, sedation scores, and complications associated with the anesthetic events using common drug protocols are summarized in Table 1 and Table 2. Premedication drugs were administered either intravenously (9%, IV), intramuscularly (78%, IM), or the route of administration was not reported (13%). The most commonly used drug combination for all anesthetic events was an α_2_-adrenergic agonist, midazolam, and ketamine (Group α_2_−adrenergic agonist, midazolam, ketamine, AMK; *n =* 34, 53%). In this group, medetomidine was the most commonly used α_2_-adrenergic agonist (76%), followed by dexmedetomidine (18%) and detomidine (6%). Administration of an α_2_-adrenergic agonist, a μ-opioid receptor agonist, and ketamine (Group α_2_−adrenergic agonist, μ-opioid receptor agonist, ketamine, AOK) occurred 13 times (20%). Medetomidine (46%) and dexmedetomidine (46%) were the most common α_2_-adrenergic agonists used with this drug combination, followed by detomidine (8%). Morphine was the most common opioid used with this drug combination (62%) followed by methadone (23%) and hydromorphone (15%). Finally, a combination of an α_2_-adrenergic agonist, midazolam, a μ-opioid receptor agonist, and ketamine (Group α_2_−adrenergic agonist, midazolam, μ-opioid receptor agonist, ketamine, AMOK) was used 10 times (16%). For this drug combination, dexmedetomidine and hydromorphone were most commonly used (50%), while dexmedetomidine was administered with morphine one time (10%). Medetomidine and morphine were used twice (20%) while hydromorphone (10%) and methadone (10%) were combined with medetomidine one time each. The remaining 11% of anesthetic events involved an alternative premedication drug protocol. For all tortoises, regardless of the premedication protocol utilized, the median (IQR) time to effect was 62 min (47–90) 

Dosages of various α_2_-adrenergic agonists and ketamine were compared between Aldabra, Galapagos, and African spurred tortoises, as these two classes of drugs were included in the anesthetic protocols of most tortoises. This comparison supported the null hypothesis that dosages of drugs administered were not significantly different between species. Medetomidine was the only α_2_-adrenergic agonist administered to Aldabra tortoises, and dosages administered to this species (0.05, 0.02–0.1 mg/kg) were not significantly different compared with dosages used for Galapagos tortoises (0.05, 0.02–0.2 mg/kg; *p* = 0.69). Medetomidine was administered one time to an African spurred tortoise at a dosage of 0.05 mg/kg. Dosages of dexmedetomidine administered to Galapagos tortoises (0.03, 0.02–0.06 mg/kg) were similar to dosages administered to African spurred tortoises (0.03, 0.02–0.05 mg/kg). Ketamine dosages between different tortoise species were not significantly different (Aldabra 4.6, 2–10.3 mg/kg, Galapagos 4.2, 1.2–10 mg/kg, African spurred 4.2, 2–9.6 mg/kg; *p* = 0.99). Power analysis calculated from the study population, assuming a clinically significant difference in dosages between the species (α 0.05) of 5 mg/kg or greater for ketamine, 0.03 mg/kg or greater for medetomidine, and 0.02 mg/kg for dexmedetomidine indicated a power of 0.79 for comparing ketamine dosages between species, 0.87 for comparing medetomidine dosages, and 0.94 for comparing dexmedetomidine dosages.

Sedation scores assigned by the anesthetist following administration of anesthetic agents included no effect, mild, moderate, and profound. These scores were subjectively determined by the individual anesthetist rather than a structured sedation scoring system. Distribution of anesthetist-assigned sedation scores for different anesthetic protocols are described in Table 2. The score of profound was only assigned to protocols in the AMK group, and eight of those nine protocols were used for Galapagos tortoises, with the other for an Aldabra tortoise.

Additional premedication or anesthetic agents were required in 25 of 64 (39%) cases to improve the quality of sedation or facilitate endotracheal intubation. Of those 25 cases, seven tortoises required sevoflurane via face mask (2–5% sevoflurane based on the vaporizer setting) to facilitate endotracheal intubation. Additional injectable drugs were required in 18 cases. Of those 18 cases, 10 required a second premedication dose, five were administered a third, and three required four or more injections. The drugs used for supplementary anesthesia by IM injection included ketamine (2.5, 0.1–5.0 mg/kg), medetomidine (0.04, 0.02–0.07 mg/kg), midazolam (0.2, 0.1–0.2 mg/kg), dexmedetomidine (0.03, 0.02–0.03 mg/kg) and tiletamine–zolazepam (2.1, 1.5–2.5 mg/kg). Anesthetic agents administered IV following an IM premedication included propofol (1.1, 0.7–1.6 mg/kg), propofol (1.0, 0.9–1.4 mg/kg) with ketamine (0.3, 0.09–1.0 mg/kg), and ketamine alone (5 mg/kg). Only one tortoise (5.5%) in group AOK needed additional injectable anesthetic agents, three (17%) tortoises in group AMOK, 10 (56%) in group AMK, and four (22%) after administration of another premedication protocol. There was no significant difference between the dosages of either α_2_-adrenergic agonist, opioid, or ketamine administered as premedication agents between these groups. All Aldabra, 11 Galapagos, and one African spurred tortoise required additional premedication agents beyond the initial premedication drugs. Table 3 illustrates which protocols required additional injections or inhalant anesthesia for each species. A total of 48 animals were intubated and the median (IQR) time to intubation for all tortoises was 80 (42–129) min.

Inhalant anesthetics (isoflurane 48%, sevoflurane 52%) were administered to 44 of 48 (92%) intubated tortoises. Nitrous oxide was administered to three animals (7%) in addition to oxygen as the carrier gas, and the remainder were administered only oxygen. Supplemental drugs administered IV to maintain an appropriate anesthetic depth were required for eight tortoises. These included either a single injection (0.05–1.14 mg/kg; *n* = 6), or a constant rate infusion (CRI) of propofol (0.05–0.1 mg/kg/hour; *n* = 1), a ketamine bolus (0.8–3.18 mg/kg; *n* = 3), or midazolam bolus (0.8 mg/kg; *n* = 1). Additional analgesics were administered to seven tortoises, including meloxicam (0.1–0.3 mg/kg; *n* = 3; subcutaneously [SQ] or IM), hydromorphone (0.02 mg/kg; *n* = 1, IM), or morphine (0.2 mg/kg; *n* = 2, IV). Infiltration with local anesthetics was performed twice; once with lidocaine (0.3 mg/kg) and once with a combination of lidocaine (0.95 mg/kg) and bupivacaine (0.1 mg/kg). Coccygeal intrathecal administration of medication was performed in two tortoises: once with lidocaine (0.26 mg/kg) and once with morphine alone (0.01 mg/kg). Pharmacologic intervention for bradycardia, hypoventilation, or apnea that occurred during general anesthesia was administered to six animals, including antagonist drugs (atipamezole 0.1 mg/kg, *n* = 2, flumazenil 0.002 mg/kg, *n* = 1), atropine (0.02–0.03 mg/kg, *n* = 4), glycopyrrolate (0.008 mg/kg, *n* = 1), and epinephrine (0.08 mg/kg, *n* = 1). 

Monitoring was performed using a variety of different multivariable monitors and included capnography, doppler assessment of heart rate (HR, Parks Medical Electronics, Beaverton, OR, USA), electrocardiography, pulse oximetry, visual assessment of respiratory rate (RR), and temperature monitoring with either an infrared thermometer gun (Raytek, Santa Cruz, CA, USA) or an esophageal temperature probe (Smiths Medical, Minneapolis, MN, USA; Philips, Cambridge, MA, USA). Thermal support was provided by the use of a heat lamp or warming blanket (Bair Hugger^TM^, 3M, St. Paul, MN, USA). Monitoring results at the start of anesthesia were evaluated. Across all species and protocols HR was 16 ± 8 bpm, spontaneous RR was 6 ± 4 bpm, manual ventilation was 6 ± 4 bpm, end-tidal carbon dioxide tension was 27 ± 9 mmHg, and body temperature was 25 ± 3 °C. Manual ventilation was performed for 24 (38%) tortoises, five ventilated spontaneously (8%), and 21 had a combination of both (33%). Ventilation was not reported in six (9%) cases, and eight tortoises were not intubated (12%).

Reversal agents, including atipamezole, flumazenil, and naloxone were administered at the beginning of the recovery period in 50 cases, not administered in three cases, and not reported in 11. Atipamezole was administered 48 times (0.17, 0.004–0.5 mg/kg). Flumazenil was administered 33 times (0.01, 0.001–0.01 mg/kg), and naloxone three times (0.01, 0.003–0.02 mg/kg). Reversals were administered IM except in two tortoises where atipamezole was administered IV. Epinephrine (0.03, 0.0008–0.1 mg/kg) was administered during recovery 10 times across nine cases and was administered IV once, intraosseous (IO) once and IM eight times. For all anesthetic events, the duration of anesthesia ranged from 45 to 405 min (125 [80–185] min) and time to extubation ranged from 7 to 225 min (58 [30–73.5] min). There were four outliers (3 Aldabra, 1 Galapagos tortoise) in this data set that had significantly longer times to extubation than the other tortoises (range 101–225 min). There were no consistent differences in clinical condition, anesthetic protocols, or anesthetic duration between these outliers versus animals with more rapid recoveries. All of the tortoises in this outlier group were administered reversal agents in the recovery period and none of these tortoises were administered epinephrine. In the recovery period, ventilation was noted to be supported manually in 22/64 (34%) anesthetic events while temperature support was noted in 3/64 (5%) events.

Reported complications were noted in 18 out of 64 total anesthetic events (28%), and included prolonged recovery (seven tortoises, 11%), apnea (seven tortoises, 11%), bradycardia (three tortoises, 5%), hypothermia (two tortoises, 3%), endotracheal tube obstruction (one tortoise, 2%), expectoration of blood and mucus (one tortoise, 2%), hypocapnia (one tortoise, 2%), and hypoventilation (one tortoise, 2%). The distribution of complications in the most commonly used protocols are outlined in Table 2. These complications were subjectively determined by the anesthesia team involved in each individual anesthetic event. There were no mortality events during the anesthetic period in this population of tortoises.

## 4. Discussion

The majority of premedication protocols used in the giant tortoises under study involved combinations of ketamine and an α_2_-adrenergic agonist. These premedication drugs were then co-administered with either a benzodiazepine alone or with a μ-opioid receptor agonist. The dosages of medetomidine and ketamine used in the tortoises were lower than dosages described in previous studies [1,3,6]. This is likely due to the clinical opinion that there would be synergistic effects of co-administered anesthetic drugs, allowing lower dosages of ketamine and α_2_-adrenergic agonists. Previous reports of midazolam administration with an α_2_-adrenergic agonist and ketamine in chelonians resulted in a reduction in all drug dosages, less cardiac depression, and shorter recoveries compared with use of α_2_-adrenergic agonists and ketamine alone [7]. The opioid dosages reported in this study were also lower than those that have been published in previous studies [4,8]. This may be due to the clinical perception of differences in species, patient size, the existence of comorbidities in this population of tortoises, or the co-administration of multiple different premedication agents permitting the use of lower dosages to achieve an adequate anesthetic plane. It is also possible that as tortoise body mass increases, metabolism decreases, therefore, necessitating lower dosages to achieve therapeutic results [9]. Also, these previous studies described dosages in relatively healthy populations of tortoises, while all the tortoises under study in this review were anesthetized to address underlying medical concerns, which may have motivated anesthetists to use lower dosages of premedication agents.

The sedation score following initial premedication was subjectively assigned by the individual anesthetists, and inter-individual variation in scoring is expected. Additionally, a standardized scoring system was not used and several of the sedation categories likely overlapped. The score of profound was only reported for AMK protocols, which was the most frequently used protocol. This may have contributed to more variation in sedation scores associated with this protocol. Also, AMK was used most frequently for Galapagos tortoises, and eight of the nine profound scores were assigned to this species. It is possible that Galapagos tortoises are more sensitive to the effects of this anesthetic protocol, contributing to a higher frequency of profound sedation. 

Both isoflurane and sevoflurane were used to successfully maintain anesthesia in the majority of giant tortoises. A lack of difference in recovery variables using inhalants of varying blood–gas solubility has been previously reported in other reptiles, including Dumeril’s monitors (*Varanus dumerilii)* and green iguanas (*Iguana iguana)* [10,11]. There are several factors beyond inhalant solubility that can influence the speed of anesthetic recovery from inhalant anesthetics in chelonians. This includes anesthetic duration and maintenance dose, co-administration of other premedication or anesthetic agents, the use of reversal agents, body temperature, minute ventilation rate in the recovery period, and the degree of right-to-left cardiac shunting that delays the development of an appropriate inhalant partial pressure within the functional lung unit to facilitate anesthetic wash-out [12,13,14]. Pulmonary shunting can be reduced or eliminated by parenteral administration of atropine or epinephrine, which can, respectively, significantly reduce the delivered concentration of isoflurane required to maintain general anesthesia, and hasten the return of spontaneous ventilation, spontaneous movement, and extubation times during recovery from inhalant agents in chelonians [12,13,15]. 

No mortalities were reported in this review and all complications were easily addressed with either reversal of anesthetic drugs, intubation and ventilation, or thermal support. The majority of tortoises required ventilatory support during the anesthetic period and into recovery, and apnea was one of the most commonly reported complications. Respiratory depression is a known sequela of anesthesia with α_2_-adrenergic agonists and μ-opioid receptor agonists in mammals and in reptiles [8,16,17,18]. Hypoventilation has been reported in desert tortoises (*Gopherus agassizii*) following medetomidine sedation, and IV administration of medetomidine and ketamine induced moderate hypoventilation in gopher tortoises (*Gopherus polyphemus)* [6,16]. These anesthetic agents were used in this population of tortoises, and likely contributed to hypoventilation and apnea. However, there are other factors influencing ventilation requirements in anesthetized tortoises, including impairment of muscular motion of the limbs that normally facilitates movement of gases through the respiratory system and the use of high concentrations of oxygen as a carrier gas during inhalant anesthesia, which may suppress the ventilatory drive of chelonians [5,7,19,20,21]. Body temperature and positioning also have a profound effect on ventilation in reptiles [22,23]. In freshwater turtles (*Chrysemas picta bellii)*, pulmonary ventilation and oxygen uptake both increased along with a rise in body temperature [19]. Variations in ambient and patient temperature may have also contributed to hypoventilation and apnea observed in the present report. 

Additional premedication drugs were necessary to achieve an appropriate plane of sedation or anesthesia in several tortoises. Fewer animals required supplemental drugs in the AOK group compared with either AMK or AMOK groups, but similar dosages were used for the individual drugs across the different premedication protocol groups. A myriad of other factors exist that contribute to the ability of injectable anesthetics to achieve a desired plane of anesthesia in chelonians, including route and location of drug administration, ambient and body temperature, the degree of activity and excitation prior to drug administration, the body condition of the patient, body position during anesthesia, and the overall clinical status of the patient [7,23]. Due to the retrospective nature of this review, these factors could not be controlled. 

This study had several limitations. Complications and sedation scores reported here were assigned by individual anesthetists and were not always clearly defined or assigned based on the same criteria. Data collected during anesthesia could not be standardized across anesthetic events, due to the retrospective nature of this study; consequently, information such as body temperature was often omitted. Due to these omissions, more in-depth statistical analysis of the data, such as factors affecting time to recovery, were not performed. Analysis was also impacted by the small sample size for sulcata tortoises. This study was slightly underpowered, especially to detect subtle differences in ketamine dosing between the species. However, there are many other factors influencing the dosage of ketamine beyond species differences, including other medications administered, health status of the animal, and body temperature. In addition, this review relied on anesthetic records from a single referral veterinary hospital, where the majority of the animals included in the study were clinically ill or injured. Therefore, information gained from this study may not translate to a healthy population. Pharmacokinetic and pharmacodynamic studies on anesthetic drugs are warranted to better elucidate their clinical effects in giant tortoises.

## 5. Conclusions

Anesthesia of Galapagos, Aldabra, or African spurred tortoises was safe and effective with any of the drug combinations reported here. A combination of an α_2_-adrenergic agonist, midazolam, and ketamine was the most common induction protocol. No mortalities were reported in this review and all complications were resolved using appropriate interventions.

## Figures and Tables

**Table 1:** Individual drugs used for immobilization of Galapagos (*Chelonoidis nigra)*, Aldabra (*Aldabrachelys gigantea)*, and African spurred tortoises (*Centrochelys sulcata)*, including the number of times each agent was administered out of 64 anesthetic events and the average dosage used (reported as mean with the range in parenthesis).

Drug	Number of Times Used	Dosage (mg/kg)
Ketamine	61	3.7 (1.24–10)
Midazolam	50	0.2 (0.06–0.5)
Medetomidine	39	0.04 (0.01–0.08)
Dexmedetomidine	18	0.02 (0.015–0.05)
Morphine	12	0.4 (0.05–0.88)
Hydromorphone	9	0.2 (0.024–0.26)
Methadone	4	0.2 (0.19–0.33)
Detomidine	3	0.04 (0.014–0.08)
Alfaxalone	1	2.0

**Table 2:** Summary of data from the three most commonly used drug class combinations used for immobilizing Galapagos (*Chelonoidis nigra;* Gal), Aldabra (*Aldabrachelys gigantea,* Ald), and African spurred tortoises (*Centrochelys sulcata;* Sul). The three most common drug class combinations were an α_2_-adrenergic agonist combined with a μ-opioid and ketamine, an α_2_-adrenergic agonist combined with midazolam and ketamine, and an α_2_-adrenergic agonist combined with a μ-opioid, midazolam, and ketamine. Sedation scores were subjectively determined by the individual anesthetist rather than a structured sedation scoring system. Data for time is reported as mean (range). α_2_, α_2_-agonist; Iso, isoflurane; Ket, ketamine; Midaz, midazolam; Mod, moderate sedation; NE, no sedative effect; N_2_O, nitrous oxide; NR, not reported; Prof, profound sedation; Sevo, sevoflurane.

Protocol	Species (*n*)	Anesthetic Events	Time to Effect (Minutes)	Sedation Score (*n*)	Time to Intubation (Minutes)	Inhalation Agent (*n*)	Anesthetic Duration (Minutes)	Time to Extubation (Minutes)	Complications (*n*)
α_2_, μ-opioid, ket	Ald (5) Gal (7) Sul (1)	13	80 (50–205)	NE (4) Mild (5) Mod (4) Prof (0)	104.5 (43–232)	Iso (3) Sevo (10)	170.2 (45–338) (170.2)	61.8 (30–105)	Prolonged recovery (2); Bradycardia (1); Apnea (1); Hypoventilation (1)
α_2_, midaz, ket	Ald (8) Gal (25) Sul (1)	34	57.6 (15–103)	NE (1) Mild (5) Mod (8) Prof (9) NR (11)	72.8 (20–150)	Iso (12) Sevo (8) N2O (2) NR (13)	123.2 (50–405) (123.2)	49.1 (0–225)	Prolonged recovery (3); Bradycardia (1); Hypothermia (2); Hypocapnia (1); Hypoventilation (1); Apnea (4); Hemoptysis (1)
α_2_, μ-opioid, midaz, ket	Ald (2) Gal (3) Sul (5)	10	79.2 (45–103)	Mild (3) Mod (4) Prof (0) NR (3)	125 (90–160)	Iso (2) Sevo (4) N2O (1) NR (2) None (1)	157.2 (47–340) (157.2)	69.7 (30–101)	Prolonged recovery (2); Bradycardia (1); Apnea (1); ETT occlusion (1)

**Table 3:** Summary of additional premedication or anesthetic agents administered to each species by the most commonly used drug class combinations. The three most common drug class combinations were an α_2_-adrenergic agonist combined with a μ-opioid and ketamine (AOK), an α_2_-adrenergic agonist combined with midazolam and ketamine (AMK), and an α_2_-adrenergic agonist combined with a μ-opioid, midazolam, and ketamine (AMOK).

Species	Protocol	Number That Received Additional Injectable Drugs	Number That Received Sevoflurane via Face Mask	Number That Did Not Receive Supplemental Anesthetic Drugs
Galapagos	AMK	6	1	19
	AOK	1	6	0
	AMOK	1	0	2
Aldabra	AMK	4	0	4
	AOK	0	0	5
	AMOK	1	0	1
Sulcata	AMK	0	0	1
	AOK	0	0	1
	AMOK	1	0	4

## Data Availability

The data presented in this study are included in this article and Supplementary Table S1.

## Supplementary Materials

The following are available online at https://www.mdpi.com/article/10.3390/ani11102920/s1, Table S1: Anesthetic drug combinations used in Galapagos (*Chelonoidis nigra*; Gal), Aldabra (*Aldabrachelys gigantea*; Ald), and African spurred tortoises (*Centrochelys sulcata*; Sul), including the dose ranges and average dose used, the species they were used in, the effect (NR: not reported; Mod: moderate; Prof: profound), time to effect, and reported complications. Drugs used include medetomidine (Med), morphine (Morph), ketamine (Ket), midazolam (Midaz), methadone (Meth), detomidine (Detom), dexmedetomidine (Dex), hydromorphone (Hydro), and alfaxalone (Alfax). Drug dosages and time to effect are reported as a range and mean.
