# Supplementary material for: Sedation and Anesthesia of Galapagos (Chelonoidis nigra), Aldabra (Aldabrachelys gigantea), and African Spurred Tortoises (Centrochelys sulcata): A Retrospective Review (2009–2019)
Source: Animals (Basel). 2021 Oct 9;11(10):2920. doi: 10.3390/ani11102920 (PMC8532946; doi:10.3390/ani11102920)
Supplement: Supplementary file 1 [file animals-11-02920-s001.zip › animals-1396766-supplementary.pdf]

animals

MDPI

Supplementary Material: Anesthetic drug combinations used in Galapagos (*Chelonoidis nigra*; Gal), Aldabra (*Aldabrachelys gigantea*; Ald), and African spurred tortoises (*Centrochelys sulcata*; Sul), including the dose ranges and average dose used, the species they were used in, the effect (NR: not reported; Mod: moderate; Prof: profound), time to effect, and reported complications. Drugs used include medetomidine (Med), morphine (Morph), ketamine (Ket), midazolam (Midaz), methadone (Meth), detomidine (Detom), dexmedetomidine (Dex), hydromorphone (Hydro), and alfaxalone (Alfax). Drug dosages and time to effect are reported as a range and mean.

Drug combination	Times used	Dosage (mg/kg)	Species	Effect	Time to Effect (minutes)	Complications
Med, Morph, Ket	4	Med: 0.02-0.048 (0.034)<br>Morph: 0.35-0.48 (0.42)<br>Ket: 2-2.14 (2.06)	Ald (4)	Mod (2 Ald)<br>NR (2 Ald)	60-205 (118.3)	Prolonged recovery (1); apnea (1)
Med, Midaz, Ket	26	Med: 0.02-0.08 (0.04)<br>Midaz: 0.07-0.5 (0.25)<br>Ket: 1.97-10 (4.29)	Ald (8)<br>Gal (18)	No effect (1 Gal)<br>Mild (4; 1 Ald, 3 Gal)<br>Mod (5 Gal)<br>Prof (7; 1 Ald, 6 Gal)<br>NR (9; 6 Ald, 3 Gal)	15-103 (52)	Prolonged recovery (2); bradycardia (1); hypothermia (2); apnea (3); hemoptysis (1); hypocapnea (1); hypoventilation (1)
Med, Meth, Midaz, Ket	1	Med: 0.0196<br>Meth: 0.33<br>Midaz: 0.065<br>Ket: 2.29	Ald (1)	Mild (1 Ald)	103	None
Morph, Midaz	1	Morph: 0.88<br>Midaz: 0.44	Gal (1)	NR (1 Gal)	NR	NR
Detom, Midaz, Ket	2	Detom: 0.03-0.08 (0.055)<br>Midaz: 0.11-0.3 (0.2)<br>Ket: 2.27-5 (3.64)	Gal (2)	Mild (1 Gal)<br>Prof (1 Gal)	50-82 (66)	Apnea (1)
Med, Meth, Ket	1	Med: 0.02<br>Meth: 0.2<br>Ket: 2.4	Ald (1)	Mod (1 Ald)	62	None
Med, Morph, Midaz, Ket	2	Med: 0.0129-0.02 (0.016)<br>Morph: 0.05-0.32 (0.19)<br>Midaz: 0.08-0.1 (0.09)<br>Ket: 1.6-2.17 (1.89)	Gal (2)	Mod (2 Gal)	62-90 (76)	Endotracheal tube occlusion (1)
Dex Midaz Ket	6	Dex: 0.015-0.05 (0.0325)<br>Midaz: 0.06-0.3 (0.24)	Gal (5)<br>Sul (1)	Mod (3 Gal)<br>Prof (1 Gal)<br>NR (2; 1 Gal, 1 Sul)	41-94 (65)	Prolonged recovery (1)

		Ket: 1.86-5 (3.81)				
Dex, Hydro Midaz, Ket	5	Dex: 0.015-0.03 (0.021) Hydro: 0.2 Midaz: 0.1-0.3 (0.2) Ket: 1.8-3 (2.56)	Sul (5)	Mild (2 Sul) NR (3 Sul)	45-85 (65)	None
Med, Ket	2	Med: 0.04-0.05 (0.045) Ket: 4-5 (4.5)	Ald (1) Gal (1)	Mild (1 Ald) NR 1 (1 Gal)	NR	Prolonged recovery (1)
Med, Hydro, Midaz, Ket	1	Med: 0.055 Hydro: 0.22 Midaz: 0.33 Ket: 5.5	Ald (1)	Mod (1 Ald)	90	Prolonged recovery (1)
Hydro, Midaz, Ket	1	Hydro: 0.1 Midaz: 0.2 Ket: 2.5	Sul (1)	Mod (1 Sul)	NR	None
Med, Hydro, Ket	1	Med: 0.05 Hydro: 0.26 Ket: 9.6	Sul (1)	Mod (1 Sul)	78	None
Med, Midaz, Alfax	1	Med: 0.04 Midaz: 0.2 Alfax: 2	Gal (1)	Mod (1 Gal)	115	None
Detom, Hydro, Ket	1	Detom: 0.014 Hydro: 0.024 Ket: 5	Gal (1)	Mild (1 Gal)	NR	None
Dex Morph, Ket	4	Dex: 0.02-0.024 (0.02) Morph: 0.38-0.4 (0.39) Ket: 1.24-2.84 (2.23)	Gal (4)	Mild (3 Gal) NR (1 Gal)	50-90 (61.3)	Prolonged recovery (1); bradycardia (1); apnea (1); hypoventilation (1)
Dex, Morph, Midaz, Ket	1	Dex: 0.02 Morph: 0.4 Midaz: 0.07 Ket: 2.86	Gal (1)	Mod (1 Gal)	45	Bradycardia; apnea
Dex Meth, Ket	2	Dex: 0.02 Meth: 0.19 Ket: 2	Gal (2)	Mild (1 Gal) NR (1 Gal)	65-75 (70)	None
Midaz, Ket	1	Midaz: 0.3 Ket: 5	Gal (1)	NR (1 Gal)	NR	None
